# Prevalence of hepatitis C infection, screening and associated factors among men who have sex with men attending gay venues: a cross-sectional survey (PREVAGAY), France, 2015

**DOI:** 10.1186/s12879-019-3945-z

**Published:** 2019-04-11

**Authors:** Sophie Vaux, Stéphane Chevaliez, Leïla Saboni, Claire Sauvage, Cécile Sommen, Francis Barin, Antonio Alexandre, Marie Jauffret-Roustide, Florence Lot, Annie Velter, for the ANRS-Prevagay group, Annie Velter, Antonio Alexandre, Francis Barin, Stéphane Chevaliez, David Friboulet, Marie Jauffret-Roustide, Florence Lot, Nathalie Lydié, Gilles Peytavin, Olivier Robineau, Leila Saboni, Claire Sauvage, Cécile Sommen

**Affiliations:** 1 Department of Infectious Diseases – Santé publique France, French national public health agency, 12 rue du Val d’Osne, 94415 Saint-Maurice, Cedex France; 2 National Reference Center for Viral Hepatitis B, C and Delta, Department of Virology, Hôpital Henri Mondor, Université Paris-Est, Créteil, France; 3 INSERM U955, Créteil, France; 4 National Reference Center for HIV, Inserm U1259 & University Hospital, Tours, France; 5 Equipe Nationale d’Intervention en Prévention et Santé pour les Entreprises (ENIPSE), Paris, France; 6 Cermes 3, Inserm U988, UMR CNRS 8211, EHESS, Paris Descartes University, Paris, France

**Keywords:** Men who have sex with men, Hepatitis C virus infection, Prevalence, Time-location sampling, Screening, HIV/HCV co-infection

## Abstract

**Background:**

Over the last 20 years, Hepatitis C virus (HCV) infection prevalence has dramatically increased among HIV-infected men who have sex with men (MSM) in many countries worldwide. It is suspected that this increase is primarily driven by sexual behaviours linked to blood exposure. Monitoring these behaviours is crucial to understand the drivers of the epidemic. This study assessed the prevalence of chronic HCV infection among MSM attending gay venues and associated chronic HCV risk factors. HCV screening and associated factors were described.

**Methods:**

The cross-sectional survey PREVAGAY, based on time-location sampling, was conducted in 2015 among MSM attending gay venues in 5 French metropolitan cities. A self-administered questionnaire was completed and capillary whole blood on dried blood spots (DBS) collected. Possible factors associated with chronic HCV prevalence and with HCV screening in the previous year were investigated using Poisson regression.

**Results:**

Chronic HCV infection prevalence from DBS analysis was 0.7% [IC95%: 0.3–1.5] in the study’s 2645 participants and was 3.0% [1.5–5.8] in HIV-positive MSM. It was significantly higher in those who reported the following: (lifetime) slamming (with or without the sharing of injection equipment); (during the previous year) fisting and chemsex, unprotected anal intercourse with casual partners, using gay websites and/or of mobile-based GPS applications, and having more than 10 sexual partners.

Only 41.3% [38.2–44.5] of the participants reported HCV screening during the previous year. Screening was significantly more frequent in MSM under 30 years of age, those who were HIV-positive, those vaccinated against hepatitis B and meningococcus C, and those who reported the following (during the previous year): more than 10 sexual partners, at least one sexually transmitted infection and fisting.

**Conclusion:**

Chronic HCV infection prevalence in MSM attending gay venues was significantly higher in HIV-positive MSM and in those with risky sexual behaviours. Reflecting current screening recommendations for specific populations, previous HCV screening was more frequent in HIV-positive individuals and those with risky sexual behaviours. Nevertheless, HCV screening coverage needs to be improved in these populations. Comprehensive medical management, which combines screening and linkage to care with prevention strategies, is essential to control HCV among MSM.

## Background

Over the last 20 years, the HCV epidemic has dramatically increased in HIV-positive men who have sex with men (MSM) in the US, Asia, Australia and Europe [1–3]. It is suspected that this increase is driven by greater exposure to contaminated blood during risky sexual behaviours including unprotected anal intercourse (UAI), fisting (brachioproctic insertion), chemsex (recreational drug use before or during sex) and having a high number of sexual partners [4, 5]. A study which performed a phylogenetic analysis using data from five countries (England, the Netherlands, France, Germany and Australia) revealed the presence of international clusters among HIV-positive MSM [3]. Monitoring risky sexual behaviours in MSM is therefore crucial to better understand the drivers of HCV transmission, and to plan and evaluate prevention interventions.

France is a low endemic country for HCV infection. In 2004, the prevalence of anti-HCV antibodies in the general population was 0.84% (CI95% [0.65–1.10]). In men and MSM, it was estimated at 0.66% [CI95%: 0.45–0.96] and 0.39% [CI95%: 0.06–2.56], respectively. The prevalence of chronic HCV infection (i.e., HCV RNA +) was estimated at 0.53% [CI95%: 0.4–0.7] in the general population, with 43% being unaware of their infection [6]. Chronic HCV prevalence tended to decrease over the following years with models estimating 0.42% in the general population in 2011 [7]. In 2009, the Prevagay study estimated, for the first time, HIV seroprevalence in MSM attending gay venues in Paris (17.7% [CI95%: 15.3–20.4]) [8]. It also estimated HCV seroprevalence (HCV antibodies) (1.02 [CI95%: 0.34–1.66]), which tended to be higher among HIV-positive MSM (1.9%) than their HIV-negative counterparts (0.8%). Updating HCV prevalence data in MSM in France is necessary to describe and understand the evolution of the epidemic and the drivers of HCV transmission.

The WHO’s (World Health Organization) goal is to eliminate viral hepatitis worldwide by 2030 [9]. France hopes to eliminate it by 2025. Since 2014, several direct-acting antiviral (DAA) drugs with excellent effectiveness against hepatitis C have been approved worldwide. Safe and well-tolerated combinations of these drugs yield very high rates of sustained virological response (over 95%) [10, 11].

HCV screening is a key element of the HCV care continuum. It allows the prompt diagnosis and treatment of patients who test positive and helps them achieve viral suppression. Because of the increased risk of HCV infection in HIV-positive MSM, international and French guidelines recommend screening for HCV in this population [12–16]. Despite the importance of screening, rates of HCV screening uptake are poorly documented. The levers and barriers to screening uptake need to be investigated in greater detail in order to guide to guide decision making on the prevention actions to implement .

In 2015, *Santé publique France*, the French national public health agency, conducted a new Prevagay study (Prevagay 2015) in MSM attending gay venues in Paris and 4 other French metropolitan cities in order to update HIV prevalence estimations through improved representativeness of this population and more precise estimators. The additional objectives of this study were to estimate HBV and HCV prevalences and to describe HCV screening in this population. The methodology of this study and the HIV and HBV prevalences values have already been published [17–19].

The present study aimed to assess the prevalence of chronic HCV infection in MSM attending gay venues and associated risk factors, and to describe HCV genotypes. It also aimed to describe HCV screening in this population and associated factors.

## Methods

### Survey design and participants

Prevagay 2015 is an anonymous cross-sectional study, using two-stage time-location sampling (TLS). TLS, also called time-space sampling or venue-based sampling, is widely used to collect data from hard-to-reach populations who frequent known locations [20]. It is widely used in surveys on MSM. The principle is to recruit individuals in physical places at times when they gather there (e.g. gay bars, clubs, backrooms) [21, 22].

Prevagay 2015 was conducted between September and December 2015 in MSM attending commercial gay venues in five French metropolitan cities (Lille, Lyon, Montpellier, Nice and Paris). The choice of these cities was based both on feasibility constraints and on epidemiological criteria. A minimum number of sufficiently frequented accessible venues was needed. Because updating HIV prevalence estimations was the main objective of this study, we chose cities with different HIV epidemiological profiles based on the number of new HIV diagnoses in MSM (French regional HIV monitoring data), the number of HIV prevalence declarations (Gay and Lesbian press survey 2011), and regional alerts of increasing numbers of STI [19]. In each city, the expected sample size was evaluated using the expected HIV prevalence and the desired precision of estimates. Two types of gay venues were investigated: bars (without sex) and backrooms or saunas (where sex was possible).

During the first stage in each city, we used simple random venue-day-time sampling without replacement, with a minimum of one visit per venue. In the second stage, for each venue-day-time, MSM were selected using systematic random sampling.

A team of investigators was created for each city, led by a local staff member from the association ‘Equipe Nationale d’Intervention en Prévention et Santé pour les Entreprises’ (ENIPSE). Among other activities, this association organises disease prevention interventions in gay venues. During visits, each team recruited participants using flyers and information letters about the survey. They also estimated the number of eligible attendees during the visit and noted the number of refusals to participate in the survey. A form collecting basic sociodemographic information was offered to MSM who refused to participate.

Men at least 18 years old, who had had sex with other men in the previous year, who were able to read and speak French, and who agreed to both perform finger-prick blood self-sampling and answer an anonymous self-administered questionnaire were eligible. Electronic tablets were used for the questionnaire and missing answers were not permitted. Test results were not given to participants but they did receive a prevention kit including condoms and flyers indicating the nearest screening centre.

The methodology is described in greater detail elsewhere [19].

### Study questionnaire

The following variables were recorded: sociodemographic characteristics, previous screening tests for HIV and HCV (response: yes, no, I don’t know) and the results of these tests, sexually transmitted infection (STI) history (including gonorrhoea, syphilis, chlamydia and *Lymphogranuloma venereum*, genital warts) during the previous year, sexual behaviours in the previous year including the number of male partners and UAI with casual partners, alcohol and psychoactive drugs’ consumption during the previous year, as well as lifetime slamming practices, and previous vaccinations against hepatitis B and meningococcal C (response: yes, no, I don’t know). Venue attendance frequency was recorded by asking participants how many times they had visited the venue included in the sampling frame in the previous month.

### Laboratory testing

HCV and HBV testing were performed by the National Reference Laboratory (NRL) for Viral Hepatitis B, C and delta (based in Creteil, France) using capillary whole blood collected on dried blood spots (DBS). The protocol used for HCV antibodies and hepatitis B surface antigen (HBsAg) detection is described elsewhere [23].

Briefly, total HCV antibodies were measured from DBS using an automated third-generation enzyme immunoassay (EIA; aHCV Vitros ECi; Ortho-Clinical Diagnostics, Raritan, New Jersey). On the basis of ROC curve analysis, the optimal cut-off ratio was estimated at 0.6. Samples with ratio values above the cut-off were considered positive.

In seropositive samples, HCV RNA level was tested for using the Abbott RealTi*m*e HCV assay (Abbott Molecular, Des Plaines, Illinois). In samples containing at least 2.5 Log IU/mL of HCV RNA, HCV genotype and subtype were determined by directly sequencing a portion of the NS5B gene encoding the RNA-dependent RNA polymerase, followed by phylogenetic analysis, as described elsewhere [24].

HBsAg detection was carried out by means of a one-step manual EIA (Monolisa™ HBs Ag ULTRA, BIO-RAD, Marnes-la-Coquette, France). On the basis of ROC curve analysis, the optimal cut-off value was established at 13.6.

HIV testing was performed by the French NRL for HIV (Tours, France) with a combined immunoassay for detection of both p24 antigen and HIV antibodies (Genscreen ultra HIV Ag-Ab; Biorad), as described elsewhere [8]. All anti-HIV positive samples were confirmed by serotyping and/or western blot [25].

### Data analysis

To make inferences from the random sample to the MSM population frequenting gay venues, a sampling weight was assigned to each participant. The generalized weight share method [26] was used in analyses, taking into account the frequency of venue attendance. More details are provided elsewhere [19].

We estimated the prevalence of biological chronic HCV infection (anti-HCV antibodies + and HCV RNA +). We also estimated the prevalence of HCV screening during the previous year.

Participants who reported consuming GHB, mephedrone or crystal methamphetamine during or just before sex were considered to have practiced chemsex [27]. Participants reporting injection of psychoactive drugs during sexual encounters were considered to have practiced slamming.

The following possible associations with chronic HCV infection and HCV screening within the previous year were investigated using univariate and multivariable Poisson regression: city, place of residence, size of the city of residence, age in years (18–29, 30–44, 45 and over), place of birth, financial situation (“Comfortable - Getting by” vs. “Struggling - Soon to be in debt”), sexual identity (homosexual, bisexual, other), HIV status (diagnosed HIV-infected, HIV-infected but not diagnosed, HIV-negative). In addition, the following possible associations were also investigated, with respect to the previous year: attending bars, saunas, backrooms and outdoor meeting places, use of gay dating websites or mobile-based GPS applications, UAI with casual partners, more than 10 sexual partners, at least one STI, consumption of alcohol (more than 6 glasses per day), BDSM (bondage, discipline, sadism and masochism), fisting and chemsex; and finally, with respect to one’s lifetime, slamming (with or without the sharing of injection equipment). Vaccinations against hepatitis B and meningococcus C (declarative data) were also investigated for HCV screening.

Only explanatory variables associated with the outcome at a *P*-value of < 0.1 in univariate analyses were eligible for the multivariable models and are listed in the results. Final multivariable models were built using backward elimination. Only variables with a *P*-value < 0.05 were kept in the final multivariable models. The prevalence ratio (PR), adjusted prevalence ratio (PRa) and 95% confidence intervals (95% CI) are presented in the results section. In all analyses (univariate and multivariable) a two-sided *p*-value < 0.05 was considered significant. Collinearity between variables was tested for. To assess whether any variables in the final model were subject to confounding by variables omitted from the final model, each omitted variable was re-introduced individually. Data analyses were performed using Stata 12.2 (StataCorp, USA).

## Results

### Profile of participants

Among the 5324 men invited to participate, 2646 agreed to do so. One blood sample was not usable for HCV analysis. Exploitable data (i.e., both a blood sample and a self-administered questionnaire) were therefore available for 2645 individuals in the five cities included (participation rate: 50%): 478 (80%) in Lille, 485 (48%) in Lyon, 266 (50%) in Montpellier, 328 (50%) in Nice and 1088 (46%) in Paris. Respondent profiles according to city are described elsewhere [19].

Twenty-one percent of non-participants agreed to complete a refusal questionnaire. Their median age was 42 years and they reported being HIV positive less frequently than participants (10%).

The majority of participants were recruited in backrooms or saunas (61.8% [CI95%: 54.0–69.0]). Median age was 41 years (mean: 40.6 years; range: 18–80 years), 64.3% [60.6–67.8] had a university degree, 82.9% [80.3–85.3] were born in France, 83.6% [80.8–86.0] defined themselves as homosexual, 12.8% [10.8–15.2] bisexual, 0.8% [0.4–1.8] heterosexual while 2.8% [1.9–4.0] refused to define themselves in relation to their sexuality. With respect to sexual behaviours in the previous year, 41.8% [38.8–45.0] reported UAI with casual partners, 44.9% [41.3–48.6] had had more than 10 sexual partners (11.4% [9.6–13.6] more than 50 in the previous year), 11.2% [9.2–13.6] reported BDSM, and 16.7% [14.6–19.0] fisting with at least one casual partner. Twelve percent (12.6%) reported chemsex during the previous year [10.8–14.7]. Slamming during one’s lifetime was reported by 3.1% [2.2–4.3] and 0.5% [0.3–0.9] declared sharing syringes/needles during slamming (Table 1). HIV prevalence was 14.3% [CI95%: 12.0–17.0] among participants while HBV infection prevalence was 0.6% [CI95%: 0.3–1.3].

**Table 1:** Characteristics of participants, PREVAGAY 2015, France – weighted data

*N* = 2645	n	%	[CI95%]
Recruitment establishments			
Bars or clubs (without sex)	1283	38.2	[31.0–46.0]
Backroom, saunas (sexual relations possible)	1362	61.8	[54.0–69.0]
City of inclusion in the survey			
Lille	478	12.0	[10.1–14.3]
Lyon	485	20.5	[16.4–25.3]
Montpellier	266	4.0	[3.3–4.9]
Nice	328	14.4	[11.3–18.2]
Paris	1088	49.0	[43.8–54.2]
Age			
18–29 y	635	24.6	[20.8–28.8]
30–44 y	1043	35.3	[32.4–38.4]
45 y and more	967	40.1	[35.5–44.9]
University degree	1686	64.3	[60.6–67.8]
Born in France	2285	82.9	[80.3–85.3]
Financial situation:			
Comfortable - Getting by	1861	71.3	[68.5–74.0]
Struggling - Soon to be in debt	784	28.7	[26.0–31.5]
Place of residence:			
France: - Town < 20,000 inhab.	517	22.2	[19.5–25.0]
- Town 20,000 ≤ ≤ 100,000 inhab.	506	19.4	[16.9–22.0]
- Town > 100,000 inhab.	1519	51.5	[48.0–54.9]
Outside France	103	7.1	[5.3–9.3]
Sexual identity:			
Homosexual	2299	83.6	[80.8–86.0]
Bisexual or other	346	16.4	[14.0–19.2]
Attendance of (during the previous year):			
Venues where sexual relations are possible (saunas, backrooms, outdoor meeting places)	2188	84.6	[80.3–88.0]
Bars (without sex)	2247	73.1	[68.4–77.4]
Backrooms	1395	48.8	[43.6–54.0]
Use of gay websites and /or use of mobile-based GPS applications	1982	69.7	[66.0–73.2]
Outdoor meeting places	1801	31.2	[28.5–34.1]
UAI with casual partners (during the previous year)	1168	41.8	[38.8–45.0]
More than 10 sexual partners (during the previous year)	1278	44.9	[41.3–48.6]
At least one STI (during the previous year)	556	17.8	[15.3–20.5]
Consumption of (before or during sexual intercourse, during the previous year):			
Psychoactive products (cocaine, GBL, GHB, ecstasy, heroin, amphetamines, crystal methamphetamine ketamine, crack, mephedrone, cathinones)	706	20.8	[18.2–23.8]
Alcohol (more than 6 glasses)	1753	58.3	[54.1–62.3]
Chemsex (during the previous year)	449	12.6	[10.8–14.7]
Slamming (lifetime)			
Yes, without sharing injection equipment	73	2.5	[1.7–3.7]
Yes and sharing injection equipment	24	0.5	[0.3–0.9]
No	2548	97.0	[95.7–97.8]
Sexual Practices with casual partners (during the previous year)			
BDSM	360	11.2	[9.2–13.6]
Fisting	464	16.7	[14.6–19.0]
Hepatitis B vaccination (self-declared)			
Yes	1735	63.0	[59.9–65.9]
No	564	22.2	[19.7–25.0]
Did not know	346	14.9	[12.6–17.4]
Meningococcus C vaccination (self-declared)			
Yes	446	14.4	[12.6–16.3]
No	1041	40.3	[37.3–43.4]
Did not know	1158	45.3	[42.3–48.4]
HCV status ^a)^			
HCV + diagnosed	12	0.3	[0.1–0.6]
HCV + undiagnosed	14	0.5	[0.2–1.3]
HCV -	2619	99.3	[98.5–99.7]
HIV status ^a)^			
HIV + diagnosed	400	13.1	[10.9–15.7]
HIV + undiagnosed	33	1.2	[0.7–2.0]
HIV -	2212	85.7	[83.1–88.0]

^a)^ undiagnosed HCV or HIV: HCV or HIV status confirmed by virologic analysis after respondent reported “negative” or “not certain of being negative” or “I don’t know” to the question on “status” in the self-administered questionnaire. Diagnosed HCV or HIV: HCV or HIV status confirmed by virologic analysis after respondent reported to be HIV or HCV “positive” in the self-administered questionnaire

With regard to vaccinations, 63.0% [59.9–65.9] of the participants declared they were vaccinated against hepatitis B and 14.4% [12.6–16.3] against meningococcus C.

### Hepatitis C prevalence

Among the 2645 participants, 26 MSM tested positive for anti-HCV antibodies and for HCV RNA. Chronic HCV prevalence (anti-HCV antibodies + and HCV RNA +) was 0.7% [0.3–1.5]. In HIV-positive and HIV-negative MSM, chronic HCV prevalence was 3.0% [1.5–5.8] and 0.3% [0.1–1.6], respectively. Among all chronically HCV infected MSM, an estimated 59.6% [21.0–89.1] were co-infected with HIV. No HBV coinfection was identified in MSM with chronic HCV. Only 37.1% [12.8–70.3] of the latter were aware of their HCV status.

Chronic HCV prevalence was 10.6% [4.5–23.1] for MSM with a history of slamming, and 16.6% [3.5–52.6] and 9.4% [3.3–24.1], in MSM who reported lifetime slamming with and without the sharing of injection equipment, respectively (Table 2). Chronic HCV prevalence was 3.5% [1.8–6.7] in those who reported chemsex in the previous year and 2.9% [1.1–7.7] in those who reported fisting. It was 1.4% [0.6–3.1] in MSM who reported more than 10 sexual partners during the previous year and 3.9% [1.4–10.5] in MSM who reported more than 50. In those aged 18–29, 30–44 and over 45 years old, respectively, it was 0.3% [0.07–1.7], 1.2% [0.4–3.5] and 0.5% [0.2–1.3].

**Table 2:** Characteristics of chronic HCV-infected MSM and associated factors, Prevagay 2015, France – weighted data

	Prevalence^a)^			Univariate analysis			Multivariable analysis		
	n	%	[CI95%]	PR	[CI95%]	p	PRa	[CI95%]	p
All (*N* = 2645)	26	0.7	[0.3–1.5]						
City									
Lille	6	0.4	[0.1–1.1]	ref.					
Lyon	3	0.5	[0.08–3.0]	1.1	[0.1–8.4]	0.9			
Montpellier	3	0.3	[0.05–1.3]	0.6	[0.1–3.8]	0.6			
Nice	6	1.0	[0.3–1.9]	2.3	[0.6–9.1]	0.3			
Paris	8	0.8	[0.3–1.5]	1.9	[0.4–7.8]	0.4			
Place of birth									
France	21	0.5	[0.2–1.0]	ref.			ref.		
Outside France	5	1.8	[0.4–7.3]	3.6	[0.7–18.0]	0.1	10.5	[3.8–28.9]	0.001
Place of residence									
France:									
- Town < 20,000 inhab.	3	0.06	[0.01–0.2]	ref.			ref.		
- Town 20,000 ≤ ≤ 100,000 inhab.	8	1.5	[0.3–6.5]	27.2	[3.3–222]	0.002	16.1	[2.4–106.8]	0.004
- Town > 100,000 inhab.	14	0.8	[0.4–1.6]	13.7	[2.6–71.3]	0.002	4.7	[0.7–31.3]	0.1
Outside France	1	0.2	[0.03–1.3]	3.6	[0.3–40.6]	0.3	0.3	[0.02–6.1]	0.5
Attendance of (during the previous year):									
- Outdoor meeting places									
yes	9	0.3	[0.1–0.7]	0.3	[0.1–1.1]	0.07			
no	17	0.9	[0.4–2.1]	ref.					
- Use of gay websites or use of mobile-based GPS applications									
yes	23	1.0	[0.5–2.1]	21.5	[4.7–98.4]	< 0.001	5.2	[1.0–27.9]	< 0.05
no	3	0.05	[0.01–0.2]	ref.			ref.		
UAI with casual partners (during the previous year)									
yes	25	1.7	[0.8–3.4]	14.3	[5.0–337.6]	0.001	11.0	[1.3–95.0]	0.03
no	1	0.04	[0.006–0.3]	ref.			ref.		
More than 10 sexual partners (during the previous year)									
yes	22	1.4	[0.6–3.1]	10.0	[2.3–44.0]	0.002	3.6	[1.1–12.3]	0.04
no	4	0.1	[0.04–0.5]	ref.			ref.		
At least one STI (during the previous year)									
yes	15	1.7	[0.8–3.4]	3.2	[0.8–13.1]	0.1			
no	11	0.5	[0.2–1.6]	ref.					
Consumption of (before or during sexual intercourse, during the previous year)									
- Alcohol (more than 6 glasses per day)									
yes	16	0.4	[0.2–0.8]	0.3	[0.08–1.0]	0.05	0.3	[0.1–0.8]	0.02
no	10	1.2	[0.4–3.2]	ref.			ref.		
- Chemsex (during the previous year)									
yes	20	3.5	[1.8–6.7]	11.3	[1.9–68.2]	0.008	3.8	[1.1–12.6]	0.03
no	6	0.3	[0.06–1.6]	ref.			ref.		
Slamming (lifetime)									
yes, without sharing of injection equipment	10	9.4	[3.3–24.1]	23.2	[5.0–109]	< 0.001	6.0	[1.6–23.3]	0.009
yes and sharing of injection equipment	3	16.6	[3.5–52.6]	41.2	[6.2–271]	< 0.001	3.8	[1.5–9.7]	0.006
No	13	0.4	[0.1–1.3]	ref.			ref.		
Practices with casual partners (during the previous year)									
- BDSM									
yes	11	1.5	[0.6–3.7]	2.5	[0.7–9.0]	0.1			
no	15	0.6	[0.2–1.5]	ref.					
- Fisting									
yes	16	2.9	[1.1–7.7]	10.8	[2.7–42.9]	0.001	5.3	[2.0–14.0]	0.001
no	10	0.3	[0.1–0.7]	ref.			ref.		
HIV status^b)^									
HIV + diagnosed	20	3.2	[1.6–6.3]	9.6	[1.8–52.2]	0.009	4.5	[1.1–18.5]	0.04
HIV + undiagnosed	1	0.2	[0.03–1.5]	0.6	[0.05–7.5]	0.7	0.3	[0.01–6.6]	0.5
HIV -	5	0.3	[0.1–1.6]	ref			ref.		
Screening for hepatitis C (previous year)									
yes	12	0.3	[0.1–0.7]	0.3	[1.0–1.0]	0.06	0.2	[0.5–0.6]	0.006
no or did not know	14	1.0	[0.4–2.4]	ref			ref.		

^a)^ n and %: crude number and weighted prevalence of chronic HCV-infected MSM in the reported category. For instance, 21 chronic HCV-infected MSM reported to be born in France; the prevalence of HCV-infected MSM among those born in France is estimated to 0.5% [0.2–1.0]

^b)^ undiagnosed HCV or HIV (MSM unaware of their HCV or HIV status): HCV or HIV status confirmed by virologic analysis after respondent reported “negative” or “not certain of being negative” or “I don’t know” to the question on “status” in the self-administered questionnaire. Diagnosed HCV or HIV (MSM aware of their HCV or HIV status): HCV or HIV status confirmed by virologic analysis after respondent reported to be HIV or HCV positive in the self-administered questionnaire

In multivariable analysis (Table 2), chronic HCV prevalence was significantly higher in MSM who lived in medium-sized towns (from 20,000 to 100,000 inhabitants) than in those living in small towns (< 20,000 inhabitants) (PRa = 16.1 [2.4–106.8], *p* = 0.004), those born outside France (PRa = 10.5 [3.8–28.9], *p* = 0.001), those who reported UAI with casual partners (PRa = 11.0 [1.3–95.0], *p* = 0.03), those who used gay websites and/or mobile-based GPS application (PRa = 5.2 [1.0–27.9], *p* < 0.05), those who reported more than 10 sexual partners in the previous year (PRa = 3.6 [1.1–12.3], *p* = 0.04), those who reported lifetime slamming (with or without the sharing of injection equipment) (PRa = 3.8 [1.5–9.7], *p* = 0.006 and PRa = 6.0 [1.6–23.3], *p* = 0.009 respectively), those who practiced fisting (PRa = 5.3 [2.0–14.0], *p* = 0.001) and those who practiced chemsex (PRa = 3.8 [1.1–12.6], *p* = 0.03) (both during the previous year). Chronic HCV prevalence was also significantly higher in HIV-infected MSM aware of their HIV status (HIV + diagnosed) (PRa = 4.5 [1.1–18.5], *p* = 0.04). Chronic HCV prevalence was significantly lower in MSM who reported consuming alcohol before or during sexual intercourse during the previous year (PRa = 0.3 [0.1–0.8], *p* = 0.02) and in MSM reporting HCV screening during the previous year (PRa = 0.2 [0.5–0.6], *p* = 0.006).

The genotype was determined from 15 HCV RNA positive samples. They were distributed as follows (crude numbers): genotype 4d (*n* = 9), genotype 1a (*n* = 5), genotype 3a (*n* = 1). In the remaining 11 HCV RNA-positive MSM, the HCV RNA level was too low (< 2.5 Log IU/mL) for amplification of the NS5B-coding region.

### HCV screening

Over 40% (41.3% [CI95%: 38.2–44.5]) of the participants reported HCV screening during the previous year, 29.4% [26.6–32.4] more than 1 year before the study, while 22.3% [19.6–25.2] reported no screening during their lifetime. Seven percent [5.6–8.6] did not know whether they had ever been screened or not. Therefore, 70.7% [67.8–73.6] of the participants reported HCV screening at least once in their life.

Fifty percent (50.1%) [45.3–54.9] of the MSM who reported more than 10 sexual partners during the previous year had been screened. This percentage was 61.2% [52.7–69.1] among HIV-infected MSM aware of their HIV status, 64.3% [58.1–70.1] among those reporting at least one STI, and 54.8% [46.9–62.5] among those who reported fisting (Table 3).

**Table 3:** Characteristics of MSM declaring screening for hepatitis C during the previous year and associated factors, Prevagay 2015, France – weighted data

	Screening^a)^			Univariate analysis			Multivariable analysis		
	n	%	[CI95%]	PR	[CI95%]	p	PRa	[CI95%]	p
All (*N* = 2645)	1219	41.3	38.2–44.5						
City									
Lille	197	41.9	[35.6–48.4]	ref.					
Lyon	227	44.9	[38.3–51.6]	1.1	[0.9–1.3]	0.5			
Montpellier	136	46.8	[39.3–54.5]	1.1	[0.9–1.4]	0.3			
Nice	149	39.9	[30.3–50.4]	0.9	[0.7–1.3]	0.8			
Paris	510	39.6	[35.2–44.2]	0.9	[0.8–1.1]	0.6			
Age									
18–29 y	328	51.2	[44.4–57.9]	ref.			ref.		
30–44 y	514	43.7	[38.9–48.6]	0.9	[0.7–1.0]	0.09	0.8	[0.7–1.0]	0.017
45 y and over	377	33.2	[28.7–38.1]	0.6	[0.5–0.8]	< 0.001	0.7	[0.6–0.8]	< 0.001
University degree									
yes	820	43.6	[39.9–47.4]	1.2	[1.0–1.4]	0.04			
no	399	37.2	[32.5–42.1]	ref.					
Sexual identity									
Homosexual	1093	43.2	[39.9–46.7]	1.4	[1.0–1.8]	0.02			
Bisexual or other	126	31.5	[24.0–40.1]	ref.					
Attendance of (during the previous year):									
- Use of gay websites or of mobile-based GPS applications									
yes	1000	46.0	[42.3–49.8]	1.5	[1.3–1.8]	< 0.001			
no	219	30.5	[25.7–35.8]	ref.					
- Bar (without sex)									
yes	1079	44.9	[41.7–48.2]	1.4	[1.2–1.7]	< 0.001			
no	140	31.5	[26.0–37.5]	ref.					
- Backrooms (sexual relations possible)									
yes	706	45.2	[40.9–49.6]	1.2	[1.1–1.4]	0.008			
no	513	37.6	[33.7–41.7]	ref.					
UAI with casual partners (during the previous year)	633	49.7	[45.3–54.0]	1.4	[1.2–1.6]	< 0.001			
yes	586	35.3	[31.7–39.2]	ref.					
no									
More than 10 sexual partners (during the previous year)									
yes	691	50.1	[45.3–54.9]	1.4	[1.2–1.7]	< 0.001	1.2	[1.0–1.4]	0.01
no	528	34.2	[30.2–38.4]	ref.			ref.		
At least one STI (during the previous year)									
yes	361	64.3	[58.1–70.1]	1.8	[1.6–2.0]	< 0.001	1.4	[1.2–1.6]	< 0.001
no	858	36.3	[33.1–39.7]	ref.			ref.		
Consumption of (before or during sexual intercourse, during the previous year)									
- Alcohol (more than 6 glasses per day)									
yes	832	44.4	[40.7–48.2]	1.2	[1.0–1.4]	0.03			
no	387	37.0	[32.1–42.2]						
Chemsex (during the previous year)									
yes	259	50.5	[42.7–58.3]	1.3	[1.1–1.5]	0.008			
no	960	40.0	[36.8–43.3]	ref					
Slamming (lifetime)									
- yes, without sharing of injection equipment	43	51.7	[36.5–66.6]	1.3	[0.9–1.7]	0.1			
- yes and sharing of injection equipment	16	77.2	[45.3–93.3]	1.9	[1.4–2.6]	< 0.001			
- no	1160	40.9	[37.7–44.1]	ref					
Risky behaviours with casual partners (during the previous year)									
- BDSM									
yes	212	59.2	[51.0–66.9]	1.5	[1.3–1.8]	< 0.001			
no	1007	39.1	[35.8–42.4]	ref.					
- Fisting									
yes	275	54.8	[46.9–62.5]	1.4	[1.2–1.7]	< 0.001	1.3	[1.1–1.5]	0.004
no	944	38.6	[35.4–42.0]	ref.			ref.		
Hepatitis B vaccination (self-declared)									
yes	915	46.5	[42.8–50.2]	ref			ref		
no	224	39.6	[32.4–47.2]	0.9	[0.7–1.0]	0.1	1.0	[0.8–1.2]	0.7
did not know	80	22.1	[15.8–30.0]	0.5	[0.3–0.7]	< 0.001	0.6	[0.4–0.8]	< 0.001
Meningococcus C vaccination (self-declared)									
yes	289	63.7	[57.0–70.0]	ref.			ref		
no	431	36.8	[32.1–41.8]	0.6	[0.5–0.7]	< 0.001	0.6	[0.5–0.8]	< 0.001
Did not know	499	38.2	[34.2–42.4]	0.6	[0.5–0.7]	< 0.001	0.7	[0.6–0.8]	< 0.001
HIV status^b)^									
HIV + diagnosed	262	61.2	[52.7–69.1]	1.6	[1.3–1.9]	0.001	1.4	[1.2–1.6]	0.001
HIV + undiagnosed	12	18.5	[7.9–37.5]	0.5	[0.2–1.0]	0.06	0.5	[0.3–0.9]	0.03
HIV -	945	38.6	[35.2–42.1]	ref					

^a)^ n and %: crude number and weighted prevalence of MSM who declared screening for hepatitis C during the previous year in the reported category. For instance, 1093 MSM who defined themselves as homosexual reported HCV screening during the previous year; the prevalence of HCV screening during the previous year among the MSM who defined themselves as homosexual is estimated to be 43.2% [39.9–46.7]

^b)^ undiagnosed HCV or HIV (MSM unaware of their HCV or HIV status): HCV or HIV status confirmed by virologic analysis after respondent reported “negative” or “not certain of being negative” or “I don’t know” to the question on “status” in the self-administered questionnaire. Diagnosed HCV or HIV (MSM aware of their HCV or HIV status): HCV or HIV status confirmed by virologic analysis after respondent reported to be HIV or HCV positive in the self-administered questionnaire

In the multivariable analysis (Table 3), HCV screening during the previous year was significantly more frequent in MSM who reported (during the previous year) more than 10 sexual partners (PRa = 1.2 [1.0–1.4], *p* = 0.01), at least one STI (PRa = 1.4 [1.2–1.6], *p* < 0.001) and fisting (PRa = 1.3 [1.1–1.5], *p* = 0.004). It was also significantly more frequent in HIV-infected MSM aware of their HIV status (PRa = 1.4 [1.2–1.6], *p* = 0.001). HCV screening was lower among MSM over 30 years of age (30–44 years: PRa = 0.8 [0.7–1.0], *p* = 0.017; 45 years and older: PRa = 0.7 [0.6–0.8], *p* < 0.001), in those who did not know whether they were vaccinated against hepatitis B (PRa = 0.6 [0.4–0.8], *p* < 0.001), in those who were not vaccinated against meningococcus C (PRa = 0.6 [0.5–0.8], *p* < 0.001), and in those who did not know whether they were vaccinated against meningococcus C (PRa = 0.7 [0.6–0.8], *p* < 0.001). HCV screening was also lower in HIV-infected MSM not aware of their HIV status (PRa = 0.5 [0.3–0.9], *p* = 0.03).

## Discussion

Among chronically HCV-infected MSM in our study, 60% were co-infected with HIV. HIV/HCV co-infection is a matter of great concern because people living with HIV not on treatment have higher HCV RNA levels and experience more rapid HCV disease progression than HIV-negative individuals [28, 29]. One systematic review estimated that the seroprevalence of HIV/HCV (i.e., positive for HCV antibodies) co-infection at 6.4% (3.2–10.0) in MSM [30]. Another review estimated the range of prevalence of active HCV infection at between 5.3 and 7.3% in HIV-infected MSM and between 4.9 and 5.9% in non-injecting HIV-infected MSM [31]. Estimates from two previous international studies showed that the risk of HCV infection was four [5] to almost six times higher (odds ratio: 5.8 [4.5–7.4]) [30] in HIV-infected MSM than in their HIV-negative counterparts. In our study, prevalences were lower than these but were consistent with estimates in the United Kingdom [32]. Prevalence data are often difficult to compare because of differences in methodologies (recruitment of MSM, biological markers used (HCV RNA vs. HCV antibodies), matrices and collection support (capillary whole blood collected on DBS vs. venous whole blood in a tube)). HCV prevalences from MSM recruited in gay venues, which are places for socializing, cannot be compared with those produced from hospitalised patients, with cohorts of HIV-infected MSM or with MSM attending STI clinics.

The role of the sexual transmission of HCV identified in our study (UAI with casual partners, more than 10 sexual partners and fisting) has already been explored in the literature. HCV infections have been reported among MSM who do not report drug injection [33]. Risky sexual behaviours such as fisting and unprotected intercourse can be mucosally traumatic and may be associated with bleeding. It has been suggested that they are also associated with the risk of contracting HCV [32, 34, 35]. Whether bleeding is necessary for HCV transmission is still debatable. Some studies have identified of HCV in seminal and rectal fluids of HIV infected men and providing evidence that these fluids can mediate HCV transmission [36, 37].

The higher risk among MSM who reported lifetime slamming (with or without sharing of injection materials) may mainly be due to the use of intravenous drugs. Indeed, the association between HCV infection and a history of IDU among MSM has already been widely described [33, 38]. Among drug users, anti-HCV antibody prevalence is high (44% [CI95%: 39.6–47.9] in France in 2011) [39]. In our study, reporting chemsex during the previous year, including the consumption of drugs by routes other than injection, was also significantly associated with a higher chronic HCV prevalence. Some studies have shown that chemsex is associated with unprotected anal sex, with sex with multiple partners and with increased STI rates [4]. One hypothesis is that risky sexual behaviours, associated with potential anal or rectal trauma resulting from longer and more intense sexual encounters under the influence of drugs, could facilitate HCV transmission among MSM.

The use of gay websites and/or mobile-based GPS applications to meet sexual partners are very popular with MSM [40]. In our study, almost 70% reported using them. The increased risk of infection and the evolution of meeting practices constitute major challenges in terms of prevention interventions for these population. Higher HCV prevalence among MSM born outside France may be partly explained by HCV prevalence in the country of birth (Africa or Asia) but also by the mobility of the gay community, particularly for festive events in European countries [41]. Special vigilance is needed for these MSM. The fact that alcohol consumption was associated with a reduced risk of chronic HCV infection may be partly explained by reduced consumption of alcohol in MSM injecting psychoactive products. The reasons why MSM living in medium-sized towns (from 20,000 to 100,000 inhabitants) had a higher risk of chronic HCV infection is difficult to explain. These results need to be confirmed in other studies. Bias cannot be excluded.

Recent data show a specific epidemiological evolution in MSM in France. Data from the Dat’AIDS cohort suggest that trends in HCV epidemiology in HIV-positive MSM differ from those observed in other HIV-positive populations. From 2012 to 2016, HCV incidence for first HCV infection increased from 0.35/100 patient-years (PY) to 0.92/100 PY in HIV-positive MSM (*p* < 0.001), while the incidence in non-MSM HIV-positive patients (with other risk factors) remained stable and close to 0.1/100 PY. The incidence of HCV reinfection was higher in HIV-positive MSM than in other HIV-positive patients with the incidence remaining stable over time in both groups [42, 43].

Our results provide clues to a greater understanding of the factors associated with HCV infection among MSM in France, and consequently can inform future prevention measures to be adopted. Higher HCV prevalences in HIV-positive MSM and among those who reported multiple sexual partners underline the need to implement routine HCV screening in these populations. Screening should also be reinforced in MSM reporting mucosally-traumatic sexual practices, chemsex and lifetime slamming, as the risk of chronic HCV infection is significantly increased.

However, the low number of chronic HCV-infected MSM (*n* = 26) limits the power of our analysis. Indeed, the wide confidence intervals underline the need to be cautious about interpreting these data.

The very low number of identified HCV genotypes in our study limits the conclusions that can be drawn on circulating genotypes in this population. However, our results are consistent with those found for HIV-infected MSM in the French Dat’AIDS cohort study where incident cases were mainly infected with genotype 4 (53%) followed with genotype 1A (39%) [43].

Since 2013, several very effective, well-tolerated and safe DAA drugs have been approved worldwide [10, 11]. DAA were made available in France in 2013 for the treatment of F3-F4 fibrosis patients. In 2016, French guidelines recommended treating all HCV infected patients irrespective of fibrosis stage [13]. The WHO’s goal is to eliminate viral hepatitis worldwide by 2030 [9], while France hopes to do so by 2025. In order to achieve this, screening is essential. HCV screening offers several potential benefits for infected persons. Patients testing positive can be promptly linked to care. Behavioural changes can be made by MSM aware of their HCV status to decrease the risk for transmission to others. Early identification and treatment are also important to limit transmission among MSM.

Only 41.3% of our study participants reported HCV screening during the previous year while 70% reported lifetime HCV screening. The latter value is close to that observed among MSM in Australia (68.1%) [44] but higher than those reported in China in 2017 (41%) and in the United States in 1999 (38.8%) [45, 46]. Few studies to date have investigated HCV screening behaviours in MSM. The differences in recruitment (MSM recruited in gay venues vs. online surveys) make comparisons difficult. Since prevention messages are often disseminated in gay venues in France, it is possible that the MSM included in our study were more aware of prevention than MSM who do not frequent such venues. Consequently, the screening percentages estimated in our study may slightly overestimate those of the overall French MSM population.

European experts recommend that HIV-infected MSM at risk of contracting acute hepatitis C infection should be screened for the disease annually [14, 15]. Additional HCV testing is recommended in HIV-positive MSM who report HCV-related sexual risk behaviours (in particular BDSM practices) and in those diagnosed with other STI [47]. The American Association for the study of liver disease (AASLD) and the infectious Disease Society of American (IDSA) recommend annual HCV testing for sexually active HIV-positive MSM and more frequent testing based on the presence of high-risk sexual or drug use behaviours [16]. For HIV-negative MSM, HCV screening is recommended for MSM who report sexual risk behaviours, in particular those who are eligible for HIV Pre-Exposure Prophylaxis (PrEP) [16, 47]. French guidelines recommend HCV screening every 6 months in HIV-infected MSM, and HCV and HIV screenings every 3 months in high-risk MSM (including those practicing chemsex, those reported an STI in the previous year, those taking PrEP, and those reporting UAI with casual partners) [12]. Because HCV reinfection incidence is high among HIV-infected MSM [48], international experts recommend HCV screening in MSM with previous and resolved HCV infection [13, 16, 47].

In the present study, HCV screening was significantly more frequent in HIV-infected MSM aware of their HIV status which is in line with other studies [44, 45]. Reflecting current screening recommendations for specific populations, previous screening was also significantly higher among MSM reporting risk behaviours such as multiple sexual partners, fisting and at least one STI during the previous year. More frequent screening also highlights the awareness of some MSM of the potential for sexual transmission of HCV. In contrast, in Australia, specific sexual activities were not associated with a greater likelihood of HCV screening [44]. HCV screening is still inadequate in France, since almost 40% of the HIV-infected MSM aware of their HIV status in our study had not been screened during the previous year. This rose to almost 50% in those who reported chemsex. Furthermore, less than 40% of chronic HCV-infected MSM were aware of their HCV status.

HCV screening in older MSM in France is also currently inadequate. Specific prevention messages and educating physicians about the importance of HCV screening in this population are vital.

Our results also highlight the importance of comprehensive medical management of MSM, which couples the actions of screening with strategies of prevention. In our study, MSM not vaccinated against hepatitis B or meningococcal C were less likely to be screened for HCV. These values were lower still among those who were unaware of their hepatitis B vaccination status, and among HIV-infected MSM unaware of their infection. General practitioners have an important role in counselling and promoting prevention actions. Expanding accessible access irrespective of risk behaviours and risk awareness of MSM is essential. MSM more exposed to at-risk situations are more likely to attend health centres which offer both screening and vaccinations [49]. MSM with a lower risk perception are less likely to feel concerned by recommendations specific to MSM [17]. Special efforts are therefore needed to reach MSM further away from care, as they may constitute low-noise virus circulation sources.

The implementation of PrEP in France could be a real opportunity for HCV prevention in HIV-negative MSM and more particularly chemsexers [12]. PrEP offers comprehensive medical monitoring included vaccinations (hepatitis A and B) and frequent testing for STI and HCV.

The use of a sampling frame for the first time to investigate HCV, HBV and HIV prevalence in MSM attending gay venues constitutes the main strength of our study. More specifically, TLS associated with sampling weights which takes account probabilities of inclusion and frequencies venue attendance in inference is very important to provide the most accurate estimations possible for MSM attending gay venues [50, 51]. Collaboration with the association ENIPSE was decisive in setting up this study. Thanks to ENIPSE’s long history, we were able to identify gay venues and gain easier access to their managers with a view to inviting them to participate. The MSM participation rate in our study was higher than or similar to those reported in other studies [21]. This may be partly explained by the fact that the investigators themselves belonged to the MSM community. Furthermore, all investigators were specifically trained to implement the survey.

The study has limitations. The use of DBS for HCV antibody detection may have led to a slight underestimation of HCV prevalence [52]. However, DBS are sensitive and specific enough to detect HCV antibodies by means of third EIA after extraction using a new cutoff ratio for interpretation [23, 53]. Although no commercial HCV RNA assay based on automated real-time polymerase chain reaction or transcription-mediated amplification has yet been approved for use with whole blood recovered from DBS, in an updated systematic review on HCV-RNA testing, pooled sensitivity and specificity values were 98% [95–99%] and 98% [95–100%] [53]. As only HCV antibody positive samples were tested for RNA, HCV antibody negative may have been missed for some very recent infections.

Among people who refused to participate, only 21% agreed to complete a refusal questionnaire. Accordingly, it was difficult to compare respondents with non-respondents. It is likely that only the most highly motivated men, for whom prevention is important, agreed to participate, and this most probably led to an underestimation of HCV prevalence and overestimation of HCV screening in our population [19].

We also need to be cautious when interpreting vaccination coverage and previous HCV screening. Self-reporting vaccination status may be associated with poor understanding and recall bias. Participants may also not correctly remember previous HCV screening (recall bias) or may not have been informed of previous screening by providers (ascertainment bias).

Finally, despite TLS being the current method of choice to conduct surveys among hard-to-reach populations such as MSM, our results cannot be extended to all the MSM population. Men recruited through TLS appeared more connected to the gay community than those recruited through internet sampling. The former also reported a greater number of sexual partners and less UAI with regular or casual partners [54].

## Conclusion

Chronic HCV prevalence in MSM attending gay venues in 5 French metropolitan cities was low but was significantly greater in HIV-infected MSM and those with risky sexual behaviours including slamming, chemsex, fisting, multiple sexual partners and UAI with casual partners. Despite current French and international recommendations, HCV screening is inadequate in this population and needs to be improved, especially among HIV-infected MSM and other MSM most exposed to risk. A fuller understanding of the drivers of the epidemic and of HCV screening are needed to identify effective public health control strategies. Comprehensive medical management, which combines the actions of screening and linkage to care with prevention strategies is essential to control HCV among MSM.

## Abbreviations

BDSM: Bondage, discipline, sadism and masochism
DBS: Dry blood spot
HBV: Hepatitis B virus
HCV: Hepatitis C virus
HIV: Human immunodeficiency virus
MSM: Men who have sex with men
NRL: National Reference Laboratory
Prep: HIV Pre-Exposure prophylaxis
PY: Patient-year
RNA: Ribonucleic acid
STI: Sexually transmitted infection
TLS: Time-location sampling
UAI: Unprotected anal intercourse
